# Microperimetry in Age-Related Macular Degeneration: An Evidence-Base for Pattern Deviation Probability Analysis in Microperimetry

**DOI:** 10.1167/tvst.8.6.48

**Published:** 2019-12-31

**Authors:** Nicola K. Cassels, John M. Wild, Tom H. Margrain, Chris Blyth, Victor Chong, Jennifer H. Acton

**Affiliations:** 1 College of Biological and Life Sciences, Cardiff University, Cardiff, UK; 2 Department of Ophthalmology, University Hospital of Wales, Cardiff, UK; 3 Department of Ophthalmology, University of Oxford, Oxford, UK

**Keywords:** age-related macular degeneration, perimetry, microperimetry, visual fields

## Abstract

**Purpose:**

The “traffic light” color designation of differential light sensitivity used in a number of microperimeters does not encompass the conventional Total and Pattern Deviation probability analyses adopted by standard automated perimetry. We determined whether the color designation is indicative of abnormality as represented by the “gold standard” Pattern Deviation probability analysis.

**Methods:**

Total and Pattern Deviation probability levels, using two different methods, were derived at each of 40 stimulus locations, within 7° eccentricity, from 66 ocular healthy individuals (66 eyes) who had undergone microperimetry with the Macular Integrity Assessment microperimeter. The probability levels were applied to the corresponding fields from each of 45 individuals (45 eyes) with age-related macular degeneration (AMD) and evaluated in relation to the color designation.

**Results:**

Sensitivities designated in orange encompassed the entire range of Pattern Deviation probability levels (from normal to *P* ≤ 1%). Those designated in green were mostly normal; those in red/black generally corresponded to the ≤1% probability level.

**Conclusions:**

The green and the red/black designations are generally indicative of normal and abnormal probability values, respectively. The orange designation encompassed all probability outcomes and should not be relied upon for visual field interpretation. The evidence base indicates replacement of the color designation of sensitivity in AMD by Total Deviation and Pattern Deviation analyses.

**Translational Relevance:**

The use of Total and Pattern Deviation probability analyses is not universal in all microperimeters, and the derivation of these values indicates that color coding will lead to errors in evaluating visual field loss.

## Introduction

Microperimetry is becoming increasingly popular for the assessment of the differential light sensitivity in macular disease.^1^–^8^ The principal advantage of microperimetry compared with standard automated perimetry (SAP) is the provision of fundus tracking that adjusts the position of each stimulus location to account for fixation instability and/or for eccentric fixation, both of which are common in late stage macular disease. Additionally, the measured sensitivity at any given stimulus location is superimposed, in real time, upon the fundus image thereby providing an indication of the topographical relationship between visual function and fundal abnormality.

The clinical interpretation of SAP at each stimulus location is based upon the difference between the measured sensitivity and the corresponding age-corrected normal value. The probability of a given difference lying outside of the normal range is used to indicate abnormality. Overall loss is identified with the Total Deviation probability map and focal loss with the Pattern Deviation probability map. The Pattern Deviation map is derived from the Total Deviation map by the general height adjustment.^9^,^10^ This type of analysis is also fundamental to other types of perimetry including frequency doubling technology perimetry^11^ and short-wavelength automated perimetry.^12^,^13^

Only one commercial microperimeter, the Compass (CenterVue, Padova, Italy), uses the Total and Pattern Deviation probability analyses.^14^ The remaining microperimeters represent the measured sensitivity at each stimulus location by a continuous scale of color designation. The color designation represents the absolute value of sensitivity, but the normal value, to which the sensitivity is referenced, varies as a function of eccentricity and of age.^15^,^16^ The inference is that green, orange, and red are indicative of normal, suspect, and abnormal outcomes. This impression is reinforced by the standard color designation for the probability levels used in optical coherence tomography, for example, for peripapillary retinal nerve fiber layer and macular thicknesses, in which normality is indicated by green and abnormality at *P* ≤ 0.01 by red, respectively. It would be useful to determine the extent to which the color designation can indicate abnormality compared with that of the Pattern Deviation probability analysis.

The derivation of the Total and Pattern Deviation probability levels at each stimulus location is based upon ordinary least squares univariate regression of the measured sensitivity against age. Two different approaches have been utilized. The age-specific method generates the deviation values, corresponding to each probability level, for each year of age^17^–^20^ based upon the prediction intervals of the regression line. The central tendency method generates the deviation values by adjusting the measured sensitivity to that of either the mean^9^,^16^ or the median^21^ of the distribution at each location, using the regression coefficient. The probability levels associated with these deviations are then generated either from the prediction intervals of the compiled distribution or empirically if the distribution is non-Gaussian.

Given the comorbidity of cataract and age-related macular degeneration (AMD), the separation of overall from focal loss is essential. The omission in commercially available microperimetry of probability analyses for overall and for focal loss is of particular concern given the obvious potential of the technique in the management of macular disease. Therefore, the color designation does not separate focal loss from that due to cataract. Furthermore, a deviation at the paracentral locations of less than 1 dB from the age-corrected normal values derived by the central tendency method in SAP can account for a change in probability level from 5% to 1%.^22^ Thus, a significant loss could be overlooked when considering the absolute value of sensitivity. The use of the Mean Deviation, Pattern Standard Deviation, mean Total Deviation and mean Pattern Deviation have been applied to the visual field from patients with AMD.^23^–^25^ The indices are summary measures of the visual field and do not provide a topographical representation of the location and spatial extent of the abnormality, which is indicated by Pattern Deviation probability analysis. Knowledge of the spatial location and extent of a defect is a fundamental principle of perimetry and is of considerable importance in AMD, given the patchy nature of the visual field loss. The concept of Total and Pattern Deviation analysis has been illustrated for several clinical cases of AMD^21^,^25^ but has not been described for a larger cohort or in relation to the color designation of absolute sensitivity.

For the evaluation of visual field loss in the absence of normative values, it would be clinically useful to determine the extent to which the color designation could indicate abnormality compared with that of the “gold standard” Pattern Deviation probability analysis. The aims of the study, therefore, were twofold. Firstly, to derive, for a commercial microperimeter that uses a “traffic light” color designation, the Total and Pattern Deviation values associated with the 5%, 2%, and 1% probability levels, respectively, using both the age-specific and the central tendency methods. Secondly, to evaluate, in individuals with AMD, the correspondence between the color designation of sensitivity used by microperimetry and the Pattern Deviation probability analysis derived by the age-specific and the central tendency methods.

## Methods

The study utilized a prospective observational case series design in an institutional setting. Written informed consent was obtained from each individual prior to enrolment in the study and after explanation of the nature and possible consequences of the study. The study adhered to the tenets of the Declaration of Helsinki, and subsequent revisions, for research involving human subjects, and the protocol was prospectively approved by the Cardiff University Research Ethics and Audit Committee and by the National Health Service South East Wales Research Ethics Committee.

### Ocular Healthy Individuals

Sixty-six ocular healthy individuals were consecutively recruited on the basis of approximately equal numbers per decade of age.^26^ The individuals were recruited from members of senior citizen and religious centers in Cardiff, UK, and from administrative staff at Cardiff University.

Each individual conformed to rigid inclusion criteria comprising: refractive error ≤ 5 dioptres sphere and 3 dioptres cylinder; visual acuity of better than or equal to 0.10 logMAR (6/7.5 Snellen) for those aged up to 60 years, and better than, or equal to, 0.18 (6/9) for those aged greater than 60 years; normally reacting pupils; normal anterior segments; crystalline lens appearance by the Lens Opacities Classification System III (LOCS III) of better than or equal to grade 2 cortical, grade 2 nuclear color and opalescence and grade 1 posterior subcapsular^27^; intraocular pressures ≤ 21 mm Hg; normal optic nerve head and fundal appearances; normal visual fields; no previous or current ocular disease, trauma, or surgery including cataract extraction and intraocular lens implantation; no history of diabetes mellitus or intracerebral disorder; no systemic medication known to affect visual function; and no family history of glaucoma. One eye of each individual was selected at random for the study.

### Individuals With AMD

The case series comprised 45 consecutively presenting individuals with AMD who were attending the macular clinics at the Cardiff Eye Unit, University Hospital of Wales, Cardiff, UK, and who had volunteered to take part in the study.

The inclusion criteria for the eyes with AMD were identical to those for the ocular healthy individuals, with the exception of wider criteria for visual acuity of better than or equal to 0.60 logMAR (6/24 Snellen) and crystalline lens appearance by LOCS III of not greater than grade 3 cortical, grade 4 nuclear color and opalescence and grade 3 posterior subcapsular.^27^ Individuals with pseudophakia were excluded.

One eye of each individual was selected at random for the study. If only one eye met the eligibility criteria, then that eye was selected. The stage of AMD in the selected eye was classified according to the Beckman scale.^28^

### Clinical Data Collection

Each individual from each group was required to attend for two visits, completed within a maximum of 3 weeks. At the first visit, all individuals underwent an ophthalmic examination including, color fundus photography (Topcon 3D OCT-1000, Topcon Corp, Tokyo, Japan) and spectral domain optical coherence tomography (SD-OCT) macular volume scans (Cirrus HD-OCT, Carl Zeiss Meditec, Inc., Dublin, CA). The ocular healthy individuals additionally underwent SAP with the Humphrey Field Analyzer (HFA; Carl Zeiss Meditec, Inc.; 740i, Central 30-2 Test, SITA Fast).

At both visits, microperimetry was undertaken with the Macular Integrity and Assessment microperimeter (MAIA; CenterVue) using a custom grid comprising 40 stimulus locations, with an interstimulus separation of 2°, extending out to 7° eccentricity (Goldmann size III; 200 msec stimulus duration; 1.27 cdm^−2^ background luminance; 318 cdm^−2^ maximum stimulus luminance; 4-2 dB double reversal of threshold). Fixation loss catch trials were determined with the Heijl-Krakau blind spot technique. The upper limit of acceptability for incorrect responses to the fixation catch trials was 15%. False-positive and false-negative catch trials are not implemented on the MAIA. However, the blind spot technique in microperimetry is not equivalent to that in SAP since microperimeters incorporate fundus tracking to identify, and correct for, fixation errors. The presentation of the stimulus at the center of the blind spot in an individual with a fixation loss corrected by fundus tracking could, therefore, theoretically, be considered to represent a false-positive catch trial.

The microperimetry results from the first visit for the ocular healthy individuals and for those with AMD were discarded to reduce the influence of any perimetric learning effect.^29^

### Derivation of the Total and Pattern Deviation Values from the Group of Ocular Healthy Individuals

The measured sensitivity as a function of age was determined at each stimulus location using ordinary least squares linear regression. The assumptions of linear regression^30^ were met at each of the 40 stimulus locations. The outcome of the linear regression at each location was then validated by bootstrapping based upon 1000 replications.^31^ The bootstrapped regression coefficients and the bias-corrected and accelerated^32^ 95% confidence intervals were estimated from the bootstrap distribution for each stimulus location.

The distributions of the Total and of the Pattern Deviation values at each location were derived for each of the two methods. The Pattern Deviation values were obtained by the general height adjustment,^9^,^10^ defined as the 85th percentile of the distribution of the Total Deviations. The deviations corresponding to the probability levels at 5%, 2%, and 1% were then calculated for each method. A liberal approach to the selection of probability levels was adopted, and the 0.5% level was therefore omitted.

### Application of the Pattern Deviation Probability Analysis to the Individuals With AMD

The Pattern Deviation probability analysis for each of the two methods was separately applied to the measured sensitivity at each stimulus location for each individual with AMD, given that the visual field loss in AMD is primarily focal.^33^–^36^

### Statistical Analysis

For the ocular healthy individuals, normality was confirmed for the distributions at each stimulus location of the Total and of the Pattern Deviation values, derived by the age-specific and by the central tendency methods, using the Kolmogorov-Smirnov test.

The difference, for the ocular healthy individuals, between the age-specific and the central tendency methods in the magnitude of the Total Deviation values required for each probability level was evaluated using a repeated measures analysis of variance (ANOVA). The within-subject factors were method (age-specific or central tendency) and probability level. The between-subject factors were eccentricity and age. An identical ANOVA was undertaken for the Pattern Deviation values.

For the individuals with AMD, the difference between the age-specific and the central tendency methods in the number of stimulus locations exhibiting abnormality (at *P* ≤ 5%) by Pattern Deviation probability analysis was evaluated using a repeated-measures ANOVA. The within-subject factor was method and the between-subject factor was eccentricity.

## Results

The demographic, visual acuity, and visual field characteristics of the ocular healthy individuals and of those with AMD are shown in the Table.

**Table:** The Demographic, Visual Acuity, and Visual Field Characteristics of the Ocular Healthy Individuals and of Those With AMD

	Normal (*N* = 66)	AMD (*N* = 45)
Age, y		
Median (IQR)	43.0 (26.0 to 65.0)	80.0 (75.0 to 83.0)
Mean (SD)	45.9 (20.8)	78.6 (7.8)
Range	19 to 93	55 to 91
Gender, male/female	28/38	18/27
VA (LogMAR; Snellen)		
Median	−0.1; 6/5	0.2; 6/9
(IQR)	(−0.2; 6/4, −0.1; 6/5)	(0.1; 6/7.5, 0.3; 6/12)
Mean (SD)	−0.1; 6/5 (0.1; 6/7.5)	0.3; 6/12 (0.2; 6/9)
Range	−0.2; 6/4 to 0.18; 6/9	0; 6/6 to 0.9; 6/48
SAP		
Mean Deviation, dB		
Median (IQR)	−0.3 (−1.0 to 0.5)	—
Mean (SD)	−0.3 (2.0)	
Range	−7.3 to 1.58	
Pattern Standard Deviation, dB		
Median (IQR)	1.5 (1.3 to 1.8)	—
Mean (SD)	1.7 (0.8)	
Range	0.6 to 5.9	
Microperimetry		
Mean Sensitivity, dB		
Median (IQR)	29.8 (28.6 to 30.5)	23.0 (19.3 to 25.2)
Mean (SD)	29.5 (1.7)	21.5 (5.8)
Range	22.7 to 32.5	5.4 to 31.4

IQR, interquartile range; SD, standard deviation; VA, visual acuity.

Of the 45 individuals with AMD, 2 exhibited early, 6 intermediate, and 37 late AMD. Of the latter, 34 had neovascular and 3 had atrophic disease. All individuals with neovascular AMD had received antivascular endothelial growth factor therapy.

For the ocular healthy individuals, the regression coefficient of sensitivity against age at each stimulus location was identical to that of the bootstrapped regression coefficient (Supplementary Table S1). The 95% confidence intervals of the regression coefficient were negative at all but two locations and exhibited almost identical values to the bootstrapped 95% confidence intervals (Supplementary Table S1).

The central tendency method was referenced to the median age of the ocular healthy individuals, 43.0 years.

The distributions of both the Total and the Pattern Deviations at each of the 40 stimulus locations was Gaussian (Kolmogorov-Smirnov, range: *P* = 0.072 to *P* = 0.200) for both the age-specific and the central tendency methods.

**Figure 1:**
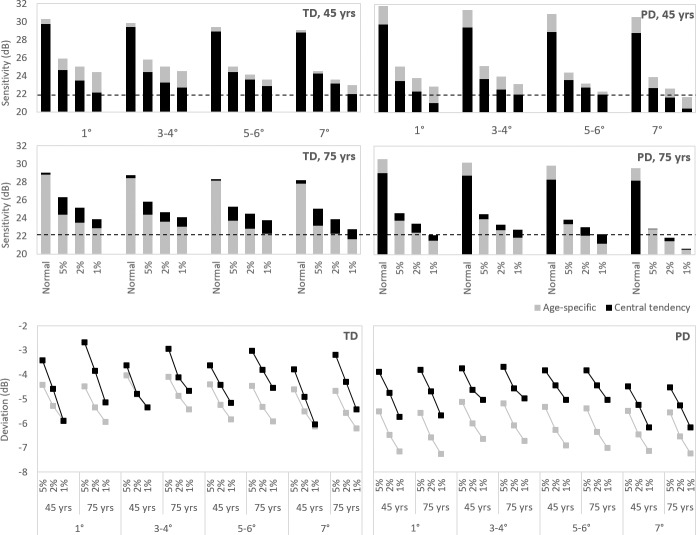
The Total and Pattern Deviation (TD and PD) values derived from the ocular healthy individuals by the age-specific (gray) and central tendency (black) methods, at each of four annuli represented by the magnitude of sensitivity and deviation from normal (top and bottom, respectively). The TD is the difference at each stimulus location between the measured sensitivity and the age-corrected normal value. The PD is the corresponding difference having corrected for any overall departure from the age-corrected normal hill of vision. (Top) The magnitude of sensitivity designated as normal and corresponding to the 5%, 2%, and 1% probability levels are shown for a 45- and a 75-year-old individual. The horizontal dashed line represents the upper limit of the sensitivity values designated in orange on the MAIA “printout.” (Bottom) The corresponding TD (left) and PD (right) values at each probability level for both ages are also shown.

The Pattern Deviation value for the designation of abnormality at each probability level was less negative for the central tendency method than for the age-specific method (*P* < 0.001) by up to 2.4 dB (Fig. 1) and was independent of age (*P* = 0.956) (Supplementary Table S2). The magnitude of the differences between the two methods, overall, became less pronounced with increasing eccentricity (*P* = 0.003). Thus, the central tendency method will detect shallower (i.e., less deep) focal abnormality compared with the age-specific method.

**Figure 2:**
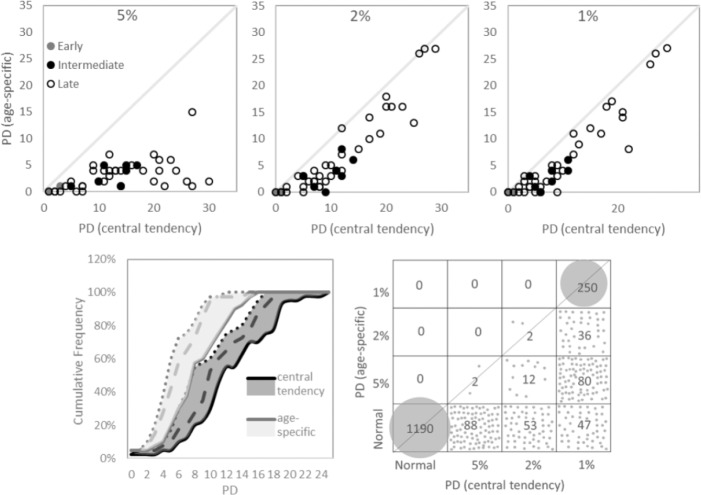
The agreement between the age-specific and central tendency methods in individuals with AMD. The agreement is shown between the number of locations exhibiting abnormality by Pattern Deviation (PD) at probability levels of 5% (top left), 2% (top middle), and 1% (top right), respectively. The cumulative frequency curves (bottom left) show the number of locations exhibiting abnormality by PD probability analysis, for each method. The number of locations exhibiting abnormality at a probability level of 5%, 2%, and 1% are shown by solid, dashed, and dotted lines, respectively (bottom left). The agreement in the PD probability level at each location, for each method is also illustrated (bottom right).

For the individuals with AMD, the difference between the age-specific and central tendency methods in the designation of visual field loss is shown in Figure 2. As expected, the number of locations exhibiting abnormality by Pattern Deviation probability analysis was greater for the central tendency method than for the age-specific method (Fig. 2) (*P* < 0.001). The difference between the two methods was most apparent at the 5% probability level and reduced as the likelihood of abnormality increased (Fig. 2, top row). At some locations, the central tendency method designated a greater likelihood of abnormality by up to three probability levels (Fig. 2, bottom right).

**Figure 3:**
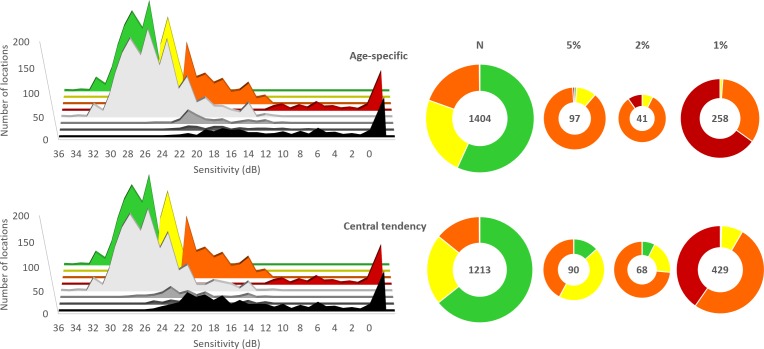
The number of locations exhibiting normality and abnormality in individuals with AMD. The left panel shows the color designation for each value of sensitivity in relation to the Pattern Deviation probability level (normal [pale gray] and P < 5% [gray], P < 2% [dark gray] and P < 1% [black]), for the age-specific (left top) and the central tendency (left bottom) methods. The pie charts show the outcome of the Pattern Deviation probability analysis defined by each method for all locations for all individuals in relation to the color designation. The corresponding number of locations is given in the center of each chart, the size of which is scaled logarithmically. N = normal.

The number of locations exhibiting abnormality by Pattern Deviation probability analysis for each method in relation to the microperimetry color designation of sensitivity is shown in Figure 3. The range of sensitivities designated in orange exhibited the greatest discrepancy (Fig. 3, right panel). Of the 478 locations exhibiting an orange designation, 170 locations (36%) were normal by the central tendency Pattern Deviation probability analysis and 220 (46%) were abnormal at the 1% probability level. Similarly, 272 (56%) of these 478 locations were designated as normal and 87 locations (18%) as abnormal at the 1% probability level by the age-specific Pattern Deviation probability analysis.

**Figure 4:**
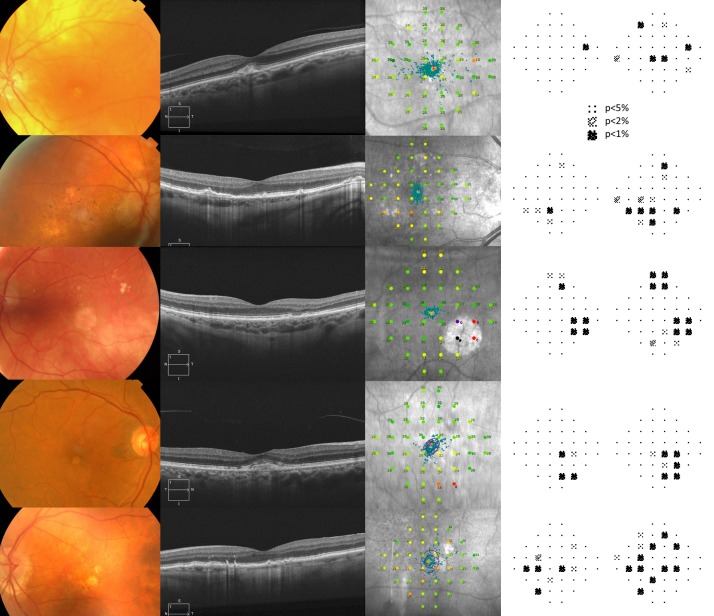
Five cases of AMD (top to bottom: aged 76, 67, 79, 74, and 80 years, respectively). Each panel from left to right shows the color fundus image, the SD-OCT horizontal line scan through the fovea, the MAIA infra-red image, and the abnormal outcomes by Pattern Deviation probability analysis using the age-specific and central tendency methods.

In 16 of the individuals with AMD, the number of locations exhibiting abnormality by Pattern Deviation probability analysis by either method was greater than the number exhibiting a color designation of orange, red, or black. Five such cases are illustrated in Figure 4.

## Discussion

This study derived Pattern Deviation probability values for microperimetry from an independently acquired group of ocular healthy individuals and applied these to a group of individuals with AMD. The purpose of the study was to evaluate the relationship between the color designation of abnormality and that defined by the Pattern Deviation probability analysis.

The key finding from this study was that the orange designation of absolute sensitivity can represent either a normal or an abnormal outcome by Pattern Deviation probability analysis. Obviously, caution should be exercised in the interpretation of sensitivity values displayed in orange. Sensitivity values designated in orange encompassed the entire range of Pattern Deviation probability levels from normal to *P* ≤ 1%, for both the age-specific and the central tendency methods. The orange designation can, therefore, indicate either normality or abnormality and is inadequate compared with a probability-based interpretation, in which the degree of certainty of abnormality is clearly defined for the clinician.

The limitations of the orange designation will be emphasized, in the absence of the general height adjustment,^10^ by the presence of diffuse loss arising from cataract. In the absence of the Total and Pattern Deviation analysis, the presence of a cataract, in an otherwise normal visual field, will shift a given color designation from green to orange or from orange to red. With focal loss in the presence of cataract, this shift will exacerbate the depth of focal loss. These limitations would lead to erroneous clinical judgement.

Those locations designated in either red or black were almost entirely associated with the *P* ≤ 1% probability level, for both methods. Those in green were almost entirely associated with normality, and this was particularly apparent for the age-specific method.

The purpose of the study was not to determine which of the two methods gave the “better” Pattern Deviation probability levels, given the absence of a reference standard. However, the central tendency method designated a greater likelihood of abnormality compared with the age-specific method. The former is the method utilized in the HFA. An evaluation of the structure-function relationship pertaining to microperimetry in AMD was also beyond the scope of the study.

The accuracy of the prediction intervals is predicated on the size of the group of ocular healthy individuals. The composition and size of the group followed that of the ISO 12866 recommendation.^26^ The adequacy of the number of ocular healthy individuals was verified by bootstrapping, which confirmed that the regression coefficients did not differ from that of a larger population. The use of bootstrapping is an accepted technique used for internal validation in predictive modelling^37^ and has been applied to confirm the validity of outcomes in studies of visual function.^38^,^39^

The Mean Sensitivity index declined with age (regression coefficient −0.04 dB/year, *R*^2^ = 0.30, *P* < 0.001). This outcome is consistent with that of −0.01 to −0.02 dB/year for microperimetry^40^–^42^ and of −0.04 to −0.08 dB/year for SAP within 10°.^15^,^17^ In addition, the ranges of sensitivity values are consistent with those reported for ocular healthy individuals^43^,^44^ and for those with AMD.^45^,^46^

A limitation of the study is that the conclusion refers to those with AMD and warrants evaluation in visual field loss arising from other disease entities. The findings from the current study apply to those with central fixation. A method of spatial interpolation both of sensitivity values and probability levels could be undertaken for individuals with eccentric fixation.^21^

The study was translational to clinical practice in that the outcome of the color coding of sensitivity was compared with the Pattern Deviation probability analysis based upon age-corrected normal values of sensitivity derived from ocular healthy individuals. The latter conformed to the inclusion criteria used for the compilation of normative values in the HFA,^47^,^48^ the Matrix perimeter,^18^ and the Octopus perimeters.^49^,^50^ An alternative design would have been to utilize a “non-AMD,” group in which the characteristics, for example, age, type, and extent of cataract, etc., would have been matched to those with AMD. The outcome of such an approach, although of scientific merit, would not have reflected clinical practice and would have limited the translational relevance of the study, as designed.

In conclusion, the findings highlight the necessity for Total and Pattern Deviation probability analyses in microperimetry, the absence of which emphasizes the inappropriate color designation of sensitivity. The evidence base indicates the following clinical recommendations for individuals with AMD for the MAIA. In AMD, sensitivity values designated in green and red/black are likely to reflect normality and true dysfunction, respectively, and those in orange should not be used to infer either normality or abnormality. For microperimeters that only employ a color designation, the significant advantage of fundus tracking is undermined by the omission of appropriate probability analyses that separate focal from overall loss.

## Supplementary Material

Supplement 1
