# Supplementary material for: Microperimetry in Age-Related Macular Degeneration: An Evidence-Base for Pattern Deviation Probability Analysis in Microperimetry
Source: Transl Vis Sci Technol. 2019 Dec 31;8(6):48. doi: 10.1167/tvst.8.6.48 (PMC6944141; doi:10.1167/tvst.8.6.48)
Supplement: Supplement 1 [file tvst-08-06-09_s01.pdf]

Supplemental Material

Supplementary Table S1

Coefficient of linear regression and 95% confidence intervals and corresponding bootstrapped outcomes.

Stimulus location	β	95% CI		Bootstrap β	95% CI	
		Lower	Upper		Lower	Upper
Location 1	-0.036	-0.062	-0.011	-0.036	-0.063	-0.009
Location 2	-0.053	-0.082	-0.023	-0.053	-0.083	-0.020
Location 3	-0.048	-0.074	-0.022	-0.048	-0.077	-0.018
Location 4	-0.043	-0.065	-0.021	-0.043	-0.065	-0.016
Location 5	-0.026	-0.053	0.001	-0.026	-0.056	0.001
Location 6	-0.044	-0.068	-0.019	-0.044	-0.062	-0.023
Location 7	-0.038	-0.061	-0.015	-0.038	-0.061	-0.011
Location 8	-0.045	-0.072	-0.019	-0.045	-0.077	-0.017
Location 9	-0.035	-0.059	-0.012	-0.035	-0.065	-0.008
Location 10	-0.043	-0.065	-0.021	-0.043	-0.062	-0.020
Location 11	-0.046	-0.070	-0.022	-0.046	-0.074	-0.018
Location 12	-0.047	-0.080	-0.013	-0.047	-0.087	-0.010
Location 13	-0.041	-0.066	-0.017	-0.041	-0.062	-0.019
Location 14	-0.049	-0.076	-0.023	-0.049	-0.076	-0.021
Location 15	-0.070	-0.094	-0.046	-0.070	-0.095	-0.045
Location 16	-0.044	-0.070	-0.018	-0.044	-0.071	-0.015
Location 17	-0.047	-0.072	-0.022	-0.047	-0.073	-0.018
Location 18	-0.037	-0.061	-0.013	-0.037	-0.057	-0.016
Location 19	-0.038	-0.071	-0.006	-0.038	-0.073	-0.002
Location 20	-0.040	-0.082	0.002	-0.040	-0.083	0.002
Location 21	-0.053	-0.085	-0.020	-0.053	-0.090	-0.013
Location 22	-0.049	-0.073	-0.025	-0.049	-0.074	-0.020
Location 23	-0.048	-0.075	-0.021	-0.048	-0.076	-0.022
Location 24	-0.051	-0.081	-0.021	-0.051	-0.090	-0.016
Location 25	-0.057	-0.082	-0.032	-0.057	-0.090	-0.025
Location 26	-0.040	-0.063	-0.016	-0.040	-0.065	-0.015
Location 27	-0.036	-0.060	-0.012	-0.036	-0.059	-0.014

Location 28	-0.049	-0.088	-0.010	-0.049	-0.083	-0.015
Location 29	-0.040	-0.063	-0.017	-0.040	-0.065	-0.013
Location 30	-0.055	-0.083	-0.027	-0.055	-0.081	-0.026
Location 31	-0.044	-0.065	-0.023	-0.044	-0.069	-0.020
Location 32	-0.051	-0.071	-0.031	-0.051	-0.070	-0.031
Location 33	-0.052	-0.078	-0.026	-0.052	-0.077	-0.023
Location 34	-0.042	-0.067	-0.017	-0.042	-0.070	-0.015
Location 35	-0.033	-0.060	-0.006	-0.033	-0.060	-0.008
Location 36	-0.041	-0.065	-0.017	-0.041	-0.073	-0.011
Location 37	-0.047	-0.076	-0.017	-0.047	-0.072	-0.019
Location 38	-0.048	-0.076	-0.021	-0.048	-0.081	-0.019
Location 39	-0.043	-0.069	-0.018	-0.043	-0.085	-0.013
Location 40	-0.036	-0.063	-0.009	-0.036	-0.062	-0.008

Supplementary Table S2

Outcomes from the repeated measures ANOVA for the difference between the age-specific and central tendency methods for the Total and for the Pattern Deviation values.

	Total Deviation		Pattern Deviation	
	F	p	F	p
Method	495.63	<0.001	438.05	<0.001
Probability Level	684.12	<0.001	1168.51	<0.001
Age	1.85	0.142	0.02	0.996
Eccentricity	6.56	<0.001	7.99	<0.001
Method*Age	14.89	<0.001	0.11	0.956
Method*Eccentricity	9.28	<0.001	3.79	0.003
Method*Probability Level	16.55	<0.001	17.90	<0.001
Probability Level*Age	0.00	1.000	0.00	1.000
Probability level*Eccentricity	5.16	<0.001	7.40	<0.001
Age*Eccentricity	0.003	1.000	0.001	1.000
Method*Probability Level*Age	0.004	1.000	0.01	1.000
Method*Age* Eccentricity	0.02	1.000	0.00	1.000
Method*Probability Level*Eccentricity	5.66	<0.001	7.45	<0.001
Probability level*Age*Eccentricity	0.00	1.000	0.00	1.000
Method*Probability Level*Age*Eccentricity	0.00	1.000	0.00	1.000
